# Neuroprotective Effect of α-Lipoic Acid against Aβ_25–35_-Induced Damage in BV2 Cells

**DOI:** 10.3390/molecules28031168

**Published:** 2023-01-24

**Authors:** Xinrong Pei, Fangyan Hu, Zehui Hu, Feiya Luo, Xiaoling Li, Shuxia Xing, Lei Sun, Dingxin Long

**Affiliations:** 1 National Institutes for Food and Drug Control, Beijing 100052, China; 2 School of Public Health, Hengyang Medical School, University of South China, Hengyang 421001, China; 3 Hunan Province Key Laboratory of Typical Environmental Pollution and Health Hazards, Hengyang Medical School, University of South China, Hengyang 421001, China

**Keywords:** Alzheimer’s disease, α-lipoic acid, neurotoxicity, microglia, Wnt/β-catenin pathway

## Abstract

The prevalence of Alzheimer’s disease (AD) is significantly increasing due to the aging world population, and the currently available drug treatments cannot cure or even slow its progression. α-lipoic acid (LA) is a biological factor widely found in spinach and meat and can dissolve in both lipid and aqueous phases. In medicine, LA has been shown to reduce the symptoms of diabetic polyneuropathy, acute kidney injury, cancers, and some metabolism-related diseases. This study to proves that α-lipoic acid (LA) can stabilize the cognitive function of patients with Alzheimer’s disease (AD). BV2 cells were divided into control, LA, Aβ_25–35_, and LA + Aβ_25–35_ groups. Cell growth; IL-6, IL-1β, TNF-α, IFN-γ, SOD, GPx, CAT, ROS, NO, and iNOS secretion; Wnt-related proteins; cell apoptosis; and cell activation were examined. Here, we found that LA could effectively repress apoptosis and changes in the morphology of microglia BV2 cells activated by Aβ_25–35_, accompanied by the inhibition of the inflammatory response induced by Aβ_25–35._ The Wnt/β-catenin pathway is also involved in preventing Aβ_25–35_-induced cytotoxicity in microglia by LA. We found an inhibitory effect of LA on microglia toxicity induced by Aβ_25–35_, suggesting that a combination of anti-inflammatory and antioxidant substances may offer a promising approach to the treatment of AD.

## 1. Introduction

Alzheimer’s disease (AD) is the most common neurodegenerative disease and seems to be one of the major healthcare challenges of the present century [1]. Approximately 50 million people worldwide had AD in 2018, and this number is expected to increase to 152 million by 2050 [2]. AD is the most common type of dementia, resulting in memory impairment and behavioral disorders [3]. It is a chronic lethal disease with a complicated pathogenesis. The two major hallmarks of AD are the formation of amyloid-β (Aβ) plaques and neurofibrillary tangles, primarily comprising the hyperphosphorylated Tau protein [4,5]. Aβ is produced and secreted by neurons in response to synaptic activity under physiological conditions. Once secreted in an extracellular environment, it is degraded by glial cells. The mechanism that causes the transition from normal physiological function to pathological Aβ accumulation is still unknown [6]. Since the currently available drug treatments cannot cure or even slow its progression [7], patients are left to rely solely on supportive care from family and other caregivers. Therefore, extensive research is necessary to investigate the molecular mechanisms of AD pathogenesis and uncover new treatment options.

**Figure 1:**
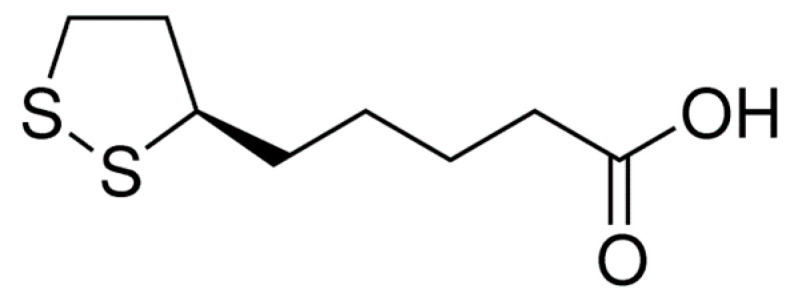
Chemical structure of α-lipoic acid (LA). The molecular formula of LA is C_8_H_14_O_2_S_2_, molecular weight is 206.326, molar refractive index is 54.94, molar volume is 169.3 m^3^/mol, and parachor (90.2 K) is 456.4.

α-lipoic acid (LA), an organosulfur medium-chain fatty acid (Figure 1) that was first discovered in 1951 as a catalytic agent for the oxidative decarboxylation of pyruvate and α-ketoglutarate [8], is a biological factor widely found in spinach, meat, and yeast and can dissolve in both lipid and aqueous phases. Healthy and young people synthesize LA naturally to scavenge reactive oxygen species (ROS) and increase endogenous antioxidants, but the level of LA significantly declines with age [9]. In medicine, LA has been shown to reduce the symptoms of diabetic polyneuropathy, acute kidney injury, cancers, and some metabolism-related diseases [10,11,12]. Previous studies have also suggested that LA has neuroprotection properties [13,14], which piqued our interest.

Microglia are an indispensable component of the central nervous system and play an important role in the nutrition, protection, and repair of neurons [15,16]. Studies have shown that microglia and AD are closely associated [17,18]. In the pathogenesis of AD, Aβ can activate microglial cells, causing them to overexpress interleukin-1, tissue growth factor (TGF)-β, and tumor necrosis factor (TNF)-α through different signal transduction pathways, as well as mediating inflammatory injury [18,19,20]. The Wnt pathways play important roles in cell activities, and Wnt dysregulation is known to be involved in Tau hyperphosphorylation and the loss of synapses [21] and neuroinflammation [22]. 

Most studies primarily focus on LA’s neuroprotective effects on neurons [18], while little is known about microglial cells. This study aimed to investigate LA’s role in Aβ_25–35_-induced microglial BV2 cell toxicity and Wnt/β-catenin signaling pathway activation.

## 2. Results

### 2.1. LA Improves Aβ_25–35_-Induced Morphology Changes and Activation in BV2 Cells

**Figure 2:**
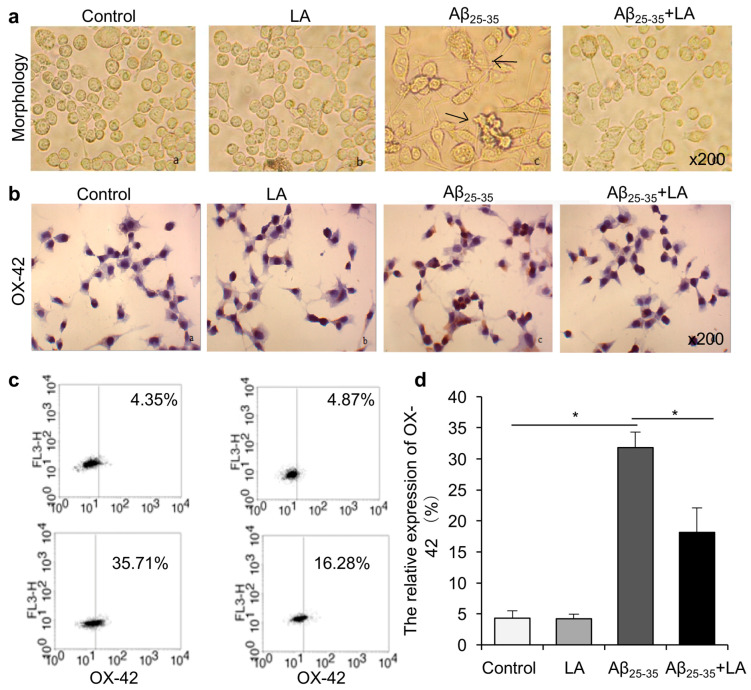
α-lipoic acid (LA) rescued Aβ_25–35_-induced BV2 cell morphology changes and inactivation. (**a**) BV2 cells were treated with 25 μmol/L Aβ_25–35_, 100 μmol/L LA, or both, and the cells were harvested for morphology observation. The changes induced by Aβ_25–35_ included larger cell bodies, cell aggregation, fusiform shape, multiple dendrites on the surface, and protrusions connecting the cells. LA attenuated the changes induced by Aβ_25–35_. Representative morphology changes are indicated by arrows. (**b**) The activation of BV2 cells was measured after different treatments. (**c**) Flow cytometry was performed to analyze the expression of OX-42. (**d**) The statistical results of the OX-42 protein expression in flow cytometry. Values are shown as mean ± SD (*n* = 3), * *p* < 0.05.

We examined the cell morphology in BV2 cells after treatment with Aβ_25–35_ to investigate whether Aβ_25–35_ treatment could induce cytotoxicity in these cells. We observed changes in cell morphology in the treated cells compared with controls, including larger cell bodies, cell aggregation, fusiform shape, multiple dendrites on the surface, and protrusions connecting the cells. We added LA to the BV2 medium before Aβ_25–35_ treatment and observed that LA could protect the cells from morphological changes induced by Aβ_25–35_. In addition, we compared these cells with Aβ_25–35_-treated cells. Decreased cell surface dendrites and changes in the shape of the cells were found in LA + Aβ_25–35_-treated cells (Figure 2a).

We next analyzed the cell activation of the BV2 cells with flow cytometry and immunohistochemistry assays. Both assays demonstrated that, compared with controls, Aβ_25–35_ treatment promoted the activation of BV2 cells. In addition, LA treatment had no significant effect on inactive cells, while it significantly repressed the activation induced by Aβ_25–35_ treatment (Figure 2b–d).

### 2.2. LA and Aβ_25–35_ Do Not Affect BV2 Cell Viability

**Figure 3:**
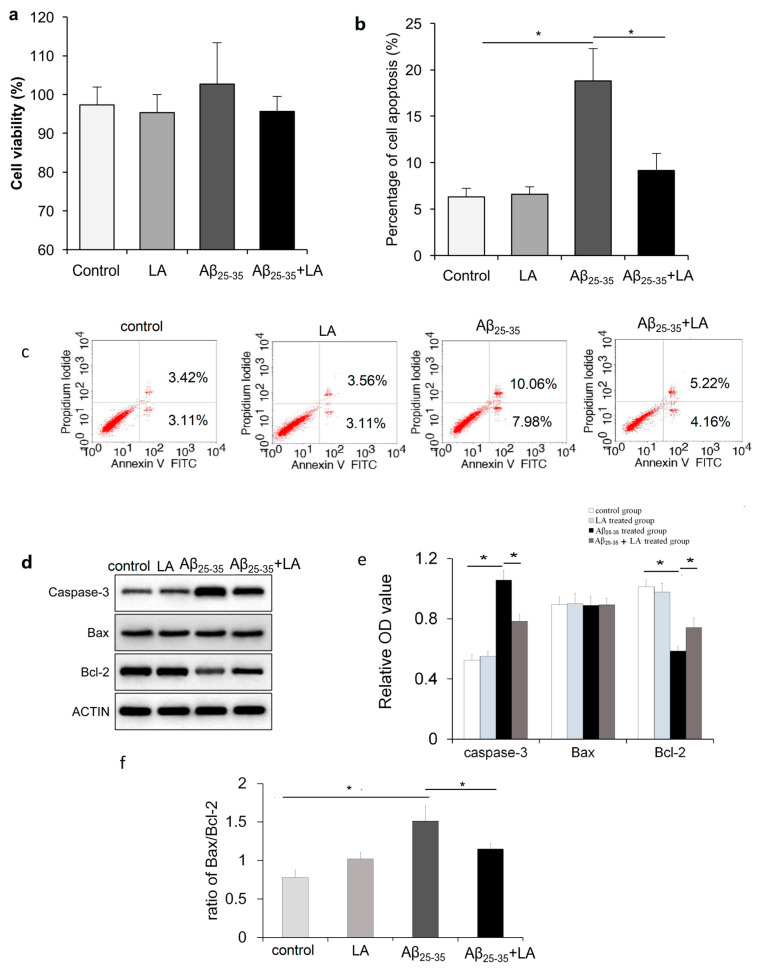
α-lipoic acid (LA) repressed Aβ_25–35_-induced BV2 cell apoptosis. (**a**) BV2 cells were treated with 25 μmol/L Aβ_25–35_, 100 μmol/L LA, or both. Cell viability was measured with the MTT assay. (**b**) Statistical analysis of the percentage of apoptosis in the different treatments. (**c**) Flow cytometric analysis of apoptosis in BV2 cells of each group. (**d**) The relative expressions of Bax, Bcl2, and caspase-3 were measured using Western blot after BV2 cells were treated with LA and/or Aβ_25–35_. (**e**) The statistical results of the protein expression in Western blot from d. (**f**) The ratio of Bax/Bcl-2 in each group. The data are presented as the mean ± SD of three independently performed experiments. * *p* < 0.05.

The cells were treated with Aβ_25–35_, which is a toxic fragment of full-length Aβ_1–42_, to investigate whether Aβ could affect the cell proliferation of BV2 cells. After 48 h, cell viability was measured by the 3-(4,5-dimethylthiazol-2-yl)-2,5-diphenyl-2*H*-tetrazolium bromide (MTT) assay. The results show that the amyloid peptide had little effect on BV2 cell proliferation compared to the control. In addition, the viability of BV2 cells remained the same, regardless of whether the cells were treated with LA alone or combined with Aβ_25–35_ (Figure 3a).

### 2.3. LA rescues Cell Apoptosis Promoted by Aβ_25–35_

Considering that Aβ_25–35_ treatment could induce BV2 cell morphology changes, using flow cytometry, we next investigated whether Aβ_25–35_ treatment promoted BV2 cell apoptosis. As shown in Figure 3, in cells treated with Aβ_25–35_, the percentage of apoptotic cells significantly increased compared to control cells. When BV2 cells were treated with Aβ_25–35_ and LA, apoptosis was largely repressed compared with Aβ_25–35_ treatment alone (Figure 3b,c). 

We also found increased protein levels of the apoptosis-related protein caspase-3, while the anti-apoptosis protein Bcl-2 was downregulated after Aβ_25–35_ treatment (Figure 3d–f) (*p* < 0.05). The expression of the Bcl-2 protein after Aβ_25–35_ treatment significantly decreased compared to the control group (*p* < 0.05). In addition, the Bax/Bcl-2 ratio of the Aβ_25–35_-treated group was significantly different from that of the control group (*p* < 0.05). After LA treatment, the abnormal expression of protein caspase-3 and Bcl-2, which were induced by Aβ_25–35_ treatment, changed. Compared with the Aβ_25–35_-treated group, the protein expression level of caspase-3 significantly decreased (*p* < 0.05), and Bcl-2 significantly increased (*p* < 0.05). In addition, the Bax/Bcl-2 ratio of the LA + Aβ_25–35_-treated group significantly decreased compared to the Aβ_25–35_-treated group (*p* < 0.05). The LA treatment alone has no significant effect on protein expression.

### 2.4. LA Is Involved in Mitigating the Inflammatory Response Induced by Aβ_25–35_

**Figure 4:**
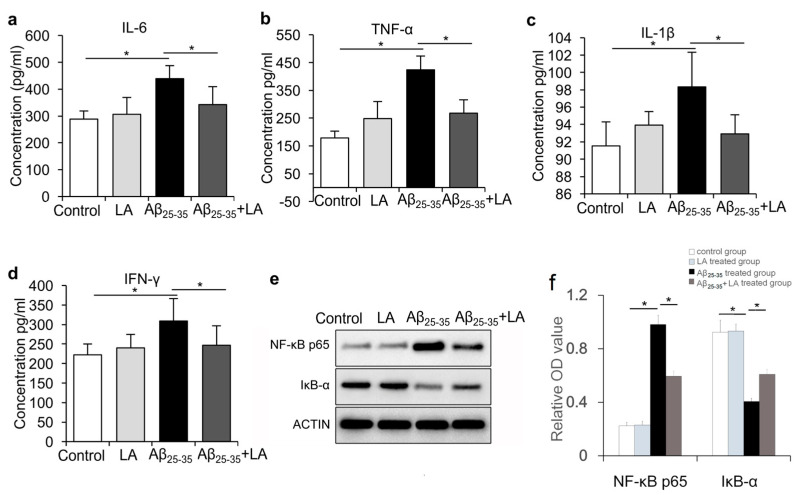
100 μmol/L α-lipoic acid (LA) inhibited the expression of inflammatory factors induced by 25 μmol/L Aβ_25–35_. (**a**–**d**) The levels of medium IL-6, IL-1β, TNF-α, and IFN-γ were measured using ELISA. (**e**) The relative expressions of NF-κB p65 and IκB-α were measured using Western blot after BV2 cells were treated with 100 μmol/L LA and/or 25 μmol/L Aβ_25–35_. (**f**) Statistical result of the protein expression in Western blot from e. The data are presented as the mean ± SD of three independently performed experiments. * *p* < 0.05.

AD pathophysiological events are usually accompanied by neuroinflammation, which is a defensive mechanism for pathogen clearance and maintenance of tissue homeostasis [23]. It has been reported that LA could reduce NF-κB activity in vitro in cells stimulated with TNF-α in a dose-dependent manner [24]. We wondered if Aβ_25–35_ treatment could induce an inflammatory response in BV2 cells and if LA would have any effect on this response. We assessed the secretion levels of IL-6, IL-1β, TNF-α, and IFN-γ via ELISA after the cells were treated with Aβ_25–35_ (Figure 4a–d). The results reveal that the expressions of IL-6, IL-1β, and TNF-α increased after Aβ_25–35_ treatment (*p* < 0.05). In addition, when we treated BV2 cells with both Aβ_25–35_ and LA, the expressions of IL-6, IL-1β, and TNF-α were repressed when compared to Aβ_25–35_ treatment alone (*p* < 0.05) (Figure 4a–d). Western blot was performed to measure the expression of NF-κB p65 and IκB-α in BV2 cells. The results show that the expression of NF-κB p65 was significantly upregulated (*p* < 0.05), while IκB-α was significantly downregulated in BV2 cells by Aβ_25–35_ treatment (*p* < 0.05). LA alone had no significant effect on NF-κB p65 and IκB-α expression. When BV2 cells were treated with both LA and Aβ_25–35_, the expression of NF-κB p65 was significantly reduced compared with Aβ_25–35_ treatment, while the expression of IκB-α significantly increased in the LA + Aβ_25–35_-treated group compared with the Aβ_25–35_-treated group (Figure 4e,f). 

**Figure 5:**
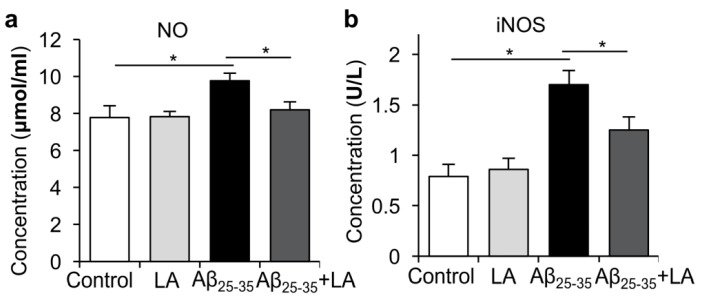
α-lipoic acid (LA) inhibited the increased level of NO and iNOS induced by Aβ_25–35_. (**a**) Statistical analysis of the release of NO after LA and/or Aβ_25–35_ treatment. (**b**) ELISA was performed to detect the activity of iNOS in BV2 cells. The data are presented as the mean ± SD of three independently performed experiments. * *p* < 0.05.

Inducible nitric oxide synthase (iNOS) is an important catalytic enzyme in organisms, which plays a biological role by catalyzing the production of nitric oxide (NO) by the substrate arginine. Innumerable studies have shown that iNOS is closely related to inflammation, and bacteria, viruses, and a variety of inflammatory factors can induce its expression to produce endogenous NO, which, in turn, plays an important biological role. Therefore, we next measured nitric oxide (NO) and inducible nitric oxide synthase (iNOS) levels in BV2 cells after Aβ_25–35_ treatment and found that NO and iNOS were increased. When we added LA to BV2 cells before Aβ_25–35_ treatment, the levels of NO and iNOS induced by Aβ_25–35_ treatment were repressed (Figure 5a,b).

### 2.5. LA Downregulates ROS Levels Induced by Aβ_25–35_

**Figure 6:**
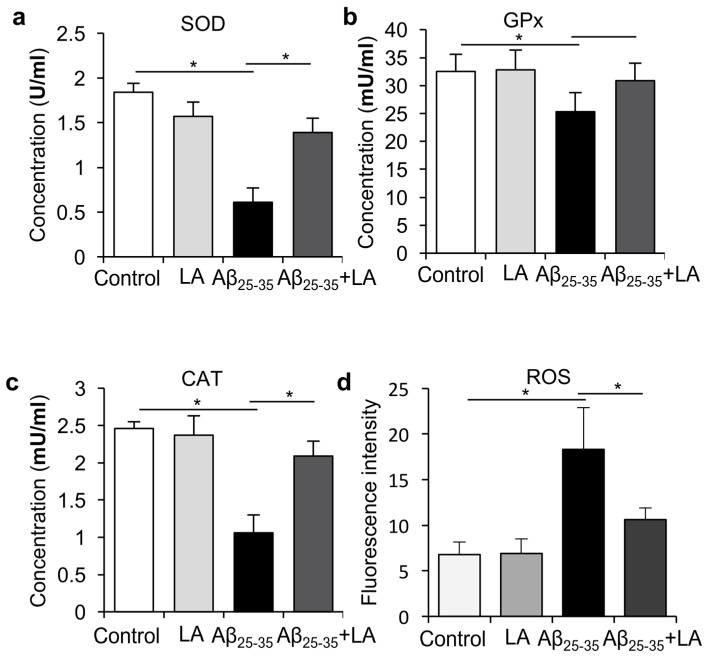
α-lipoic acid (LA) regulated the activity of SOD, GPx, CAT, and ROS in BV2 cells induced by Aβ_25–35_. (**a**–**c**) BV2 cells were treated with 25 μmol/L Aβ_25–35_, 100 μmol/L LA, or both for 24 h, and then the activities of SOD (**a**), GPx (**b**), and CAT (**c**) were detected. (**d**) The ROS levels were detected with a fluorescent probe tagged DCFH-DA after BV2 cells were treated with LA and/or Aβ_25–35_ for the indicated time. Values are shown as mean ± SD (*n* = 3), * *p* < 0.05.

Neurodegenerative disorders such as AD are associated with oxidative damage [4]. In order to investigate whether LA could modify the Aβ_25–35_-induced ROS increase, we treated BV2 cells with LA and Aβ_25–35_ to observe the activity of SOD, GPx, CAT, and ROS in BV2 cells induced by Aβ_25–35_ and LA. We found that after LA treatment, the enzyme activities of SOD, GPx, and CAT increased (Figure 6a–c), while ROS levels were significantly repressed (Figure 6d) compared to treatment with Aβ_25–35_ alone. The results demonstrate that LA could reduce the Aβ_25–35_-induced ROS levels in mouse microglia BV2 cells.

### 2.6. LA-regulated Wnt Pathway-Specific Protein Expression in Aβ_25–35_-Treated BV2 Cells

**Figure 7:**
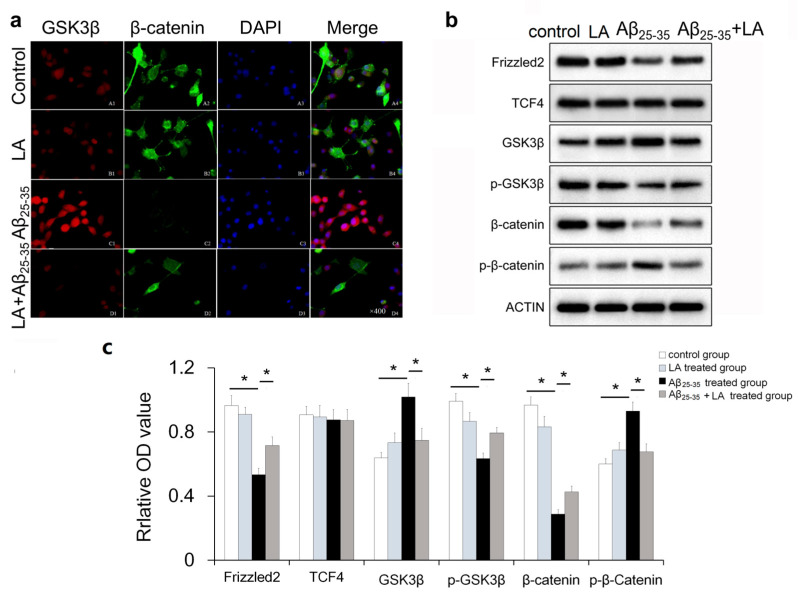
100 μmol/L α-lipoic acid (LA) rescued the inactivated Wnt pathway induced by 25 μmol/L Aβ_25–35_ in BV2 cells. (**a**) The expression and localization of GSK3β (red) and β-catenin (green) were visualized by confocal analysis (100×). (**b**) Western blot was performed to analyze the expression of Frizzled2, TCF4, GSK3β, p-GSK3β, β-catenin, and p-β-Catenin. (**c**) Statistical results of the protein expression in Western blot from b. The data are presented as the mean ± SD of three independently performed experiments. * *p* < 0.05.

It has been reported that Wnt signaling inactivation promotes the neurotoxicity of Aβ [25,26]. In order to determine whether the Wnt pathway participated in the neuroprotective role of LA, we analyzed the cellular localization and expression of GSK3β and β-catenin after Aβ_25–35_ treatment or treatment with both Aβ_25–35_ and LA (Figure 7a). The expression of GSK3β increased after Aβ_25–35_ treatment while β-catenin decreased. In addition, LA treatment upregulated β-catenin expression and inhibited the expression of GSK3β induced by Aβ_25–35_.

Western blot indicated that the expression of phosphorylated GSK3β (p-GSK3β), Frizzled2, and β-catenin was downregulated, while phosphorylated β-catenin (p-β-catenin) was upregulated in BV2 cells after Aβ_25–35_ treatment (Figure 7b,c). When BV2 cells were treated with both LA and Aβ_25–35_, the inactivated Wnt pathway was re-activated, and the associated proteins were recovered. Specifically, after Aβ_25–35_ treatment, the expressions of Frizzled2, GSK3β, p-GSK3β, β-catenin, and p-β-catenin were significantly different compared to the control group, and the expressions of Frizzled2, p-GSK3β, and β-catenin were significantly reduced compared with the control group. The expressions of GSK3β and p-β-catenin significantly increased when compared to the control group. After LA intervention, the expression of Frizzled2 and p-GSK3β in the LA + Aβ_25–35_-treated group significantly increased compared with that of the Aβ_25–35_-treated group, and the expressions of GSK3β and β-catenin in the LA + Aβ_25–35_-treated group were significantly decreased compared to the Aβ_25–35_-treated group. LA treatment has no significant effect on protein expression.

## 3. Discussion

The pathogenesis of AD is complicated, and the underlying mechanisms are not fully understood. Accumulating evidence shows that inflammation plays an important role in AD’s pathogenesis, and the deposition of Aβ can activate brain inflammation, resulting in nervous system damage [27,28,29]. Microglia, the central nervous system’s immune cells, are widely distributed in the central nervous system. Their activation promotes inflammatory responses in the brain, increasing the progression of AD [30]. The results show that BV2 cells were activated and morphologically changed after treatment with Aβ. The number of OX-42-positive cells also increased after Aβ_25–35_ treatment, indicating an increase in activated microglia. After LA intervention, the cell morphology was improved compared with the Aβ_25–35_ treatment alone. The results demonstrate that LA can effectively inhibit the Aβ-induced apoptosis of glial cells, which might be one of the important mechanisms of LA neuroprotection.

Previous studies reported that inflammatory cytokines produced by microglial cells, including IL-6 and TNF-α, play an important role in AD’s pathogenesis [6]. During AD pathogenesis, IL-6 overexpression is associated with the abnormal phosphorylation of Tau. Our study indicates that Aβ_25–35_ induced inflammatory cytokines production, including IL-6, TNF-α, and IL-1β, while the LA intervention significantly reduced their levels. In addition, LA can reduce the activation of BV6 cells induced by Aβ_25–35_, which could have important consequences on AD’s development since activated microglia are responsible for Tau hyperphosphorylation [17,18]. We also found that the expressions of endothelial nitric oxide synthase (eNOS) and iNOS were upregulated with Aβ_25–35_ treatment, and LA was able to reduce their upregulation. Low levels of NO production protect against oxidative stress, while high NO production is associated with increased damage, consistent with AD’s pathogenesis [31,32].

Previous studies have reported that the Wnt/β-catenin pathway is involved in AD’s pathogenesis, although most studies were mainly focused on neurons and less on glial cells [25,33]. Here, we found that the expression of some Wnt/β-catenin pathway proteins such as Frizzled2, GSK3β, p-GSK3β, β-catenin, and p-β-catenin was altered in the glia after Aβ_25–35_ treatment, suggesting that the Wnt pathway was also involved in Aβ_25–35_-induced glial cytotoxicity. The Wnt pathways are known to play important roles in cell activities, and Wnt dysregulation is known to be involved in Tau hyperphosphorylation, the loss of synapses [21], and neuroinflammation [22]. The already known effect of Aβ on Wnt pathways has two aspects. One is that Aβ and the amyloid precursor protein (APP) promote β-catenin phosphorylation and degradation, thus inhibiting the canonical Wnt pathway [21,34]. The tau protein is believed to stabilize b-catenin so that it can resist degradation, and the abnormal modification of tau can also cause damage to the canonical Wnt pathway [35]. The dysregulated expression of these proteins was rescued following LA intervention. The results suggest that the Wnt pathway genes are involved in LA’s neuroprotection potential in Aβ_25–35_-treated microglia cells. Further studies are needed to elucidate how LA plays its protective role through this pathway.

Taken together, the effects of LA observed here are consistent with its effects in various chronic diseases [10,11,12], as well as in nerve cells [13,14]. Of note, LA had no cytotoxicity effects on BV6, suggesting that it is not toxic for these cells. These effects seen at the microglia levels are supported by clinical observations that LA can improve patients’ outcomes with AD [36,37,38]. Nevertheless, the effects of LA in AD are controversial [39,40] and might depend upon the model used. The present study shows that the effect of LA on microglia was consistent with effects that should slow down AD’s progression, but in vivo studies remain necessary.

In conclusion, this study shows that Aβ_25–35_ inhibited BV2 cell activity and promoted cell apoptosis. After Aβ_25–35_ treatment, the Wnt pathway was inactivated, antioxidant enzyme activity was reduced, and ROS were elevated. With an LA intervention, the inflammatory reaction and apoptosis induced by Aβ_25–35_ were repressed. The results indicate that LA has a potential protective effect on nerve cells and that the Wnt/β-catenin signaling pathway is involved in the effects of LA. This study provides a theoretical basis for the application of LA in the treatment or management of AD. 

## 4. Materials and Methods

### 4.1. Cell Culture

BV2 cells were purchased from Peking Union Medical College, Chinese Academy of Medical Sciences, School of Basic Medicine Cell Center (Beijing, China). These cells were then cultured in DMEM (Hyclone, Thermo Fisher Scientific, Waltham, MA, USA) and supplemented with 10% fetal bovine serum, 100 U/mL penicillin, and 0.1 mg/mL streptomycin at 37 °C in 5% CO_2_. The medium was replaced every 2–3 days. 

### 4.2. Observation of Cell Morphology

Cells in the logarithmic growth phase were adjusted to 3 × 10^5^/mL and seeded into 6-well plates. The cells were divided into control, LA treatment, Aβ_25–35_ treatment, and LA + Aβ_25–35_ treatment groups. For LA treatment, 100 μmol/L LA (Sigma, St Louis, MO, USA) was added to the cells and incubated for 24 h. For Aβ_25–35_ treatment, 25 μmol/L Aβ_25–35_ (Sigma, St Louis, MO, USA) was added to the cells followed by 24 h culture before the cells were harvested for observation. For the LA + Aβ_25–35_ treatment group, 100 μmol/L LA was added to the cells, which were then incubated for 2 h. Then, 25 μmol/L of Aβ_25–35_ was added, and the cells continued to culture for 24 h, after which they were harvested for the observation of cell morphology.

### 4.3. Cell Growth Assays

Cell viability was measured using an MTT assay, as previously described [41]. The cells were seeded into 96-well plates and maintained in culture. After treatments according to grouping, the cells were further incubated for 48 h, washed twice with PBS, and incubated with 100 μL MTT (5 g/L) for 4 h at 37 °C. The optical densities of the solutions were measured at 570 nm. Duplicate measurements were performed in three independent wells at each time point. For the LA + Aβ_25–35_ treatment group, 100 μmol/L LA was added to the plates and incubated for 2 h before Aβ_25–35_ treatment. 

### 4.4. ELISA Detection for IL-6, IL-1β, TNF-α, and IFN-γ

Cells in the logarithmic growth phase were adjusted to 5 × 10^5^/mL and seeded into 24-well plates. The cells were treated according to grouping. The supernatant was collected for IL-6, IL-1β, TNF-α, and IFN-γ ELISA assays (R&D Systems, Minneapolis, MN, USA), according to the manufacturer’s instructions.

### 4.5. Detection of SOD, GPx, CAT, ROS, NO, and iNOS 

Cells in the logarithmic growth phase were adjusted to 5 × 10^5^/mL and seeded into 24-well plates. The cells were treated according to their grouping. The supernatant was collected for SOD, GPx, CAT, ROS, NO, and iNOS detection, according to the manufacturer’s instructions (Jiancheng Institute of Biotechnology, Nanjing, China).

### 4.6. Western Blot

The cells were washed with PBS and lysed on ice for 30 min with RIPA (Applygen Technologies Inc., Beijing, China) containing a protease inhibitor mixture (Fermentas, Burlington, ON, Canada). The total protein was subjected to 10% sodium dodecyl sulfate-polyacrylamide gel electrophoresis and was transferred to nitrocellulose membranes (Millipore Corp., Billerica, MA, USA). After blocking in 5% non-fat dry milk in TBST, the membranes were incubated with primary antibodies overnight at 4 °C. The membranes were washed three times with TBST and incubated with HRP-conjugated secondary antibodies for 1 h at room temperature. Proteins were visualized using a chemiluminescent substrate (Millipore Corp., Billerica, MA, USA) according to the manufacturer’s instructions.

### 4.7. Cell Apoptosis and Activation by Flow Cytometry

For the detection of cell activation, the cells were harvested and washed with PBS. The OX-42 antibody (Santa Cruz Biotechnology, Santa Cruz, CA, USA) was added, and the cells were incubated overnight at 4 °C. The next day, a secondary FITC-conjugated antibody (Zhongshan Biotechnologies Inc., Zhongshan, China) was added and incubated for 30 min. The cells were washed with PBS and analyzed. For the apoptosis analysis, the Annexin V/propidium iodide (PI) staining kit was used according to the manufacturer’s instructions (BioLegend, San Diego, CA, USA), and the cells were stained with FITC-conjugated with Annexin V and PI. Stained cells were examined using a FACSCanto II (FACSAria, BD Biosciences, Franklin Lake, NJ, USA). The data were analyzed using the FlowJo software 10 (BD Biosciences, Franklin Lake, NJ, USA).

### 4.8. Immunofluorescence

Immunofluorescence was performed as previously described [42]. The cells were grown on coverslips and fixed for 20 min in 4% paraformaldehyde. The cells were blocked in 5% goat serum albumin, incubated at 4 °C overnight with primary antibodies against β-catenin and GSK3 (Table 1), and then incubated with a secondary antibody: anti-Mouse IgG (H + L), F(ab’)2 Fragment (Alexa Fluor^®^ 555 Conjugate), or Anti-Rabbit IgG (H + L), F(ab’)2 Fragment (Alexa Fluor^®^ 488 Conjugate) (Abcam, Cambridge, United Kingdom). DAPI was used as a nuclear counterstain. Microscopic analyses were performed using an FV1500 confocal microscope (Olympus, Tokyo, Japan).

### 4.9. Statistical Analysis

All the data are expressed as means ± standard deviations (SD) and were analyzed using one-way analysis of variance (ANOVA) with the Student–Newman–Keuls multiple-range test using SPSS 20.0 (IBM, Armonk, NY, USA). Two-sided *p*-values < 0.05 were considered statistically significant.

## Figures and Tables

**Table 1:** Antibodies for Western blotting.

Primary Antibodies.	Dilution	Manufacturer	Secondary Antibody Dilution
Frizzled 2	1:200	Santa Cruz	1:4000
TCF4	1:1000	Abcam	1:4000
GSK3β	1:5000	Abcam	1:4000
p-GSK3β	1:10,000	Abcam	1:4000
β-catenin	1:5000	Abcam	1:4000
p-β-catenin	1:1000	CST	1:4000
IκBα	1:1000	CST	1:4000
NF-kB p65	1:200	CST	1:4000
Bcl-2	1:500	Santa Cruz	1:4000
Bax	1:300	Santa Cruz	1:4000
p-NFκB p65	1:500	CST	1:4000
Caspase-3	1:5000	Abcam	1:4000
β-actin	1:200	Santa Cruz	1:8000

Santa Cruz: Santa Cruz Biotechnology, Santa Cruz, CA, USA. Abcam: Abcam, Cambridge, United Kingdom. CST: Cell Signaling Technology, Inc., Danvers, MA, USA.

## Data Availability

Not applicable.

## Sample Availability

Samples of the compounds are available from the authors.

## Abbreviations

Alzheimer’s disease (AD), α-lipoic acid (LA), phosphorylated GSK3β (p-GSK3β), phosphorylated β-catenin (p-β-catenin), reactive oxygen species (ROS), amyloid-β (Aβ), tissue growth factor (TGF), tumor necrosis factor (TNF), nitric oxide (NO), inducible nitric oxide synthase (iNOS), endothelial nitric oxide synthase (eNOS), propidium iodide (PI), standard deviations (SD), one-way analysis of variance (ANOVA).
